# Studying Irony Detection Beyond Ironic Criticism: Let's Include Ironic Praise

**DOI:** 10.3389/fpsyg.2017.00606

**Published:** 2017-04-21

**Authors:** Richard Bruntsch, Willibald Ruch

**Affiliations:** ^1^Department of Psychology, Section Personality and Assessment, University of ZurichZurich, Switzerland; ^2^Department of Psychology, Distance Learning University SwitzerlandBrig, Switzerland

**Keywords:** cheerfulness, confirmatory factor analysis, corrective humor, intelligence, ironic praise, irony, personality, STCI

## Abstract

Studies of irony detection have commonly used ironic criticisms (i.e., mock positive evaluation of negative circumstances) as stimulus materials. Another basic type of verbal irony, ironic praise (i.e., mock negative evaluation of positive circumstances) is largely absent from studies on individuals' aptitude to detect verbal irony. However, it can be argued that ironic praise needs to be considered in order to investigate the detection of irony in the variety of its facets. To explore whether the detection ironic praise has a benefit beyond ironic criticism, three studies were conducted. In Study 1, an instrument (Test of Verbal Irony Detection Aptitude; TOVIDA) was constructed and its factorial structure was tested using *N* = 311 subjects. The TOVIDA contains 26 scenario-based items and contains two scales for the detection of ironic criticism vs. ironic praise. To validate the measurement method, the two scales of the TOVIDA were experimentally evaluated with *N* = 154 subjects in Study 2. In Study 3, *N* = 183 subjects were tested to explore personality and ability correlates of the two TOVIDA scales. Results indicate that the co-variance between the ironic TOVIDA items was organized by two inter-correlated but distinct factors: one representing ironic praise detection aptitude and one representing ironic criticism detection aptitude. Experimental validation showed that the TOVIDA items truly contain irony and that item scores reflect irony detection. Trait bad mood and benevolent humor (as a facet of the sense of humor) were found as joint correlates for both ironic criticism and ironic praise detection scores. In contrast, intelligence, trait cheerfulness, and corrective humor were found as unique correlates of ironic praise detection scores, even when statistically controlling for the aptitude to detect ironic criticism. Our results indicate that the aptitude to detect ironic praise can be seen as distinct from the aptitude to detect ironic criticism. Generating unique variance in irony detection, ironic praise can be postulated as worthwhile to include in future studies—especially when studying the role of mental ability, personality, and humor in irony detection.

## Introduction

Ironic criticism and ironic praise can be distinguished as two basic types of verbal irony (cf. Kreuz and Link, 2002). The two types are structurally similar to each other as both involve mock evaluations of circumstances with a valence opposite to the speaker's true appraisal. As the characteristic difference between the two, ironic praise is characterized by a negative valence in what is said and a positive valence in the speaker's true appraisal of circumstances while in ironic criticism the converse is true^1^.

When we use irony, we typically utter something different from what we want to express, i.e., typically the opposite of our true appraisal of circumstances. Characteristically, we expect the listener to recognize our overt dissimulation by seeing through the counterfactual nature of our utterance and to eventually detect the intended meaning of what we say nonetheless (Groeben and Scheele, 2003). However, this is not always the case, as listeners may not detect the irony for certain reasons. For example, imperfect irony detection rates were found as a function of the ambiguity of the context of ironic utterances. Accordingly, Ackerman (1983) reports considerable average error rates in his irony detection task (ranging from 5.6 to 24.1% depending on the difficulty of the stimuli) in a control group consisting of college students. Furthermore, individuals differ in their aptitude to detect verbal irony, which results in systematic variance in irony detection performance (e.g., Winner et al., 1998; see Bruntsch et al., 2016, for an overview).

In the studies investigating irony detection, a plethora of tasks and *ad-hoc* test has been used to assess individuals' aptitude to detect verbal irony. However, most of these studies did not utilize both *ironic criticism* (as a mock positive evaluation of negative circumstances) and *ironic praise* (as a mock negative evaluation of positive circumstances). Rather, the stimuli used in the existing studies on irony detection mostly rely on ironic criticisms (such as in the form of sarcasm^2^), whereas ironic praise is not represented (e.g., Ackerman, 1983; Happé, 1993; McDonald and Pearce, 1996; Mitchley et al., 1998). This is somewhat puzzling, as ironic praise can be found as counterbalanced with ironic criticism in the stimuli used in studies targeting different aspects of irony processing, such as when investigating processing times (i.e., response latencies) of ironic stimuli vs. their literal counterparts (Schwoebel et al., 2000). Likewise, there are studies investigating perceived speaker's intent in “ironic insults” (matching the definition of *ironic criticism* we adhere to; cf. Kreuz and Link, 2002) and “ironic compliments” (matching the definition of *ironic praise*) vs. direct insults and direct compliments, respectively (e.g., in terms of ratings of mocking and politeness, i.e., Pexman and Olineck, 2002).

However, studies investigating irony *detection* have largely neglected the sampling of ironic praise stimuli. This may be owed to the view that ironic praise can be seen as the less prevalent and less “prototypically ironic” type of irony (cf. Kreuz and Link, 2002). However, a study by Langdon et al. (2002) demonstrated that stimuli containing ironic praise led to different results than ironic criticism stimuli. Langdon et al. (2002) used both, ironic criticism (labeled as *sarcasm*) and ironic praise (labeled as *banter*), and distinguished them in separate scores for their investigation of irony detection in schizophrenic patients vs. normally functioning control subjects^3^. As Langdon et al. (2002) report, ironic praise was harder to detect than ironic criticism, especially in the group of patients with schizophrenia. Thus, it can be hypothesized that ironic praise may be the very type of irony that is affected by impaired or unusual cognitive and affective functioning. More generally, it may be suggested that ironic praise leads to meaningful interindividual variance in irony detection tasks beyond the one found for ironic criticism.

## Ironic criticism vs. ironic praise

As detailed below, we argue that the two types of irony can be distinguished considering at least three aspects: (a) they have different purposes and functions in communication, (b) in irony detection ironic praise may depend on individuals' expression of certain traits more than ironic criticism, and (c) in irony detection they demand different cognitive and affective processes in individuals.

(A) One may characterize that ironic praise is typically used for different purposes (for example good-natured “ironic teasing;” Keltner et al., 2001) than ironic criticism (for example aggressive ridicule). In the form of teasing, ironic praise may be reasoned to be a way to humorously apprise the recipient of social norms when harmless transgressions occur—such as when using it as a playful provocation in socializing, flirting, or entertaining. In contrast, ironic criticism may be employed for the purpose of apprising the recipient of social norms when more severe transgressions occur—such as when resolving conflicts by aggressive ridicule (cf. Norrick, 1994; Keltner et al., 2001). Furthermore, as ironic praise is typically used in the face of positive circumstances, one may reason that ironic praise is more suitable than ironic criticism (which in turn is typically used in the face of adverse circumstances) for certain of the discourse goals found for verbal irony, such as to be funny or witty, to be humorous, and to play or to be silly (cf. Kreuz et al., 1991).

(B) The different functional aspects of the two types of irony (such as different utilities in social interaction) may affect the detection of ironic criticism and ironic praise differently, depending on individuals' expression of certain traits, such as the sense of humor. As the notion that humor is a function of irony is pervasive in the literature (cf. Bruntsch et al., 2016), the sense of humor (which can be defined as relatively stable interindividual differences in the tendency to react to humor and to produce humor, and a serene attitude toward life; see Ruch, 1998) can be assumed to go along with the readiness to detect or mis-detect verbal irony. Certain *facets* of the sense of humor may come into play more evidently in the detection of ironic praise than in the detection of ironic criticism. Furthermore, looking at ironic praise as a playful and light-hearted figure of speech, its detection may be facilitated by cheerfulness (e.g., Ruch et al., 1996) more than this is the case for ironic criticism. This may be the case because highly cheerful individuals may process cues signaling playfulness more readily, which helps to reject the uttered negative evaluation and detect the more positive implication of ironic praise. Importantly, this may not hold true for ironic criticism, which may be seen as less playful and less jocular than ironic praise.

(C) It can be argued that the norm violation that irony typically alludes to and criticizes (e.g., Utsumi, 2000; Garmendia, 2014) is harder to recognize in the case of ironic praise: it may be more obvious and hence easier to understand why ironic criticism is used. This may be because people generally have positive expectations (e.g., successful players in professional sports; cf. Kreuz and Link, 2002). Thus, the detection of ironic praise may require a more complex mental representation of the background of the ironic remark and a more effortful cognitive search for the antecedent event that ironic remarks typically refer to (Kreuz and Glucksberg, 1989), as compared to the detection of ironic criticism. In line with this consideration, intelligence may be more relevant for the detection of ironic praise than for the detection of ironic criticism. If the role of intelligence truly was more evident in the detection of ironic praise, ironic praise should be included in irony research when mental abilities as well as mental impairments are targeted.

## Aims of the paper

The current paper has three main aims. Firstly, a test for the assessment of irony detection with two different scales (i.e., ironic criticism vs. ironic praise) will be developed, opting for an indirect measurement format (Study 1). It is aimed to use two testing modes with different degrees of *irony alertness*: hiding the measurement intention from participants (i.e., *irony non-alert* mode) vs. making irony salient (*irony alert* mode). Using confirmatory factor analysis, the two-factor structure (corresponding to the distinction between ironic criticism and ironic praise) will be tested. Secondly, in Study 2 we will validate the soundness of the stimuli and the indirect measurement by (a) using an experimental approach (i.e., comparing four testing conditions: irony alert testing, irony non-alert testing, forced ironic interpretation, and forced literal interpretation), (b) testing whether there is a convergence between the test scores and direct irony-ratings, and (c) comparing direct irony-ratings between ironic items and non-ironic distractor items (which should differ from each other). Thirdly, Study 3 will explore ability and personality correlates of the two scales. It is expected that ironic praise detection scores are at least as strongly related—if not even more strongly related—to (a) intelligence, (b) the ability to distinguish irony from a lie, (c) different facets of the sense of humor, and (d) traits constituting the temperamental foundation of the sense of humor (e.g., cheerfulness), as this is the case for the detection of ironic criticism.

## Study 1: development of the test of verbal irony detection aptitude (TOVIDA)

It is assumed that there is meaningful interindividual variance in irony detection performance in terms of an irony detection aptitude. It is hypothesized that this aptitude comprises two facets: the aptitude to detect ironic criticism and the aptitude to detect ironic praise. After selecting those items with the most acceptable psychometric features, a confirmatory factor analysis will be employed to investigate whether the two predefined concepts used in the instrument (ironic criticism and ironic praise) are represented by two different structural components. A first sample will be used to determine psychometric properties under *irony non-alert* testing conditions, as this unobtrusive method can be reasoned to reflect individuals' everyday mode of dealing with irony (i.e., usually, we do not deliberately watch out for irony). Then, a second sample will be used for cross-validation to see whether the fit of a two-factor model (i.e., ironic criticism vs. ironic praise) can be confirmed under *irony alert* testing conditions. Maximizing irony alertness can be reasoned to reduce systematic noise in the interindividual variance. To specify: as some individuals may be more biased not to anticipate irony in a psychological survey than others, *irony non-alert* testing presumably would lead to artificial co-variance between the items. Furthermore, as the shared variance between items systematically depends on the interindividual variance that makes co-variance arise in the first place, this method can be seen as a source of data accommodating a more conservative test of the assumed model.

### Methods

#### Participants

Participants were recruited via university mailing lists, social platforms, and leaflets. Two independent samples were used. Sample 1 consisted of 152 German-speaking subjects (40 males [35.7%]). Age in Sample 1 ranged from 18 to 51 years with a mean of 22.8 (*SD* = 5.8). Sample 2 consisted of 159 German-speaking subjects (39 males [32.5%]). Age in Sample 2 ranged from 18 to 67 years with a mean of 24.1 (*SD* = 7.3).

#### Materials

##### Test of Verbal Irony Detection Aptitude-40 (TOVIDA-40)

To develop a test for the assessment of irony detection aptitude, 30 scenarios containing ironic target utterances (among which 20 contained ironic criticism and 10 contained ironic praise) and 10 scenarios with non-ironic target utterances were written using a rational construction procedure. Irony detection was defined as the comprehension of the true meaning of ironic target utterances as opposite to the literal meaning in ambiguous situations short of distinct information. Each scenario consists of a short story about two or more people and culminates in a final utterance (the target utterance) made by one of the protagonists. Target utterances contain either verbal irony or literal speech. When generating the stimuli, irony was designed as follows: in the ironic utterances used in the *ironic criticism* stimuli, speakers (i.e., the story characters making the target utterance) use a choice of words which, when used non-ironically, denotes a positive appraisal—while ironically implying an opposite (i.e., negative) appraisal. Conversely, as the characteristic feature of the utterances found in the *ironic praise* stimuli, speakers use a choice of words which, when used non-ironically, denote a negative appraisal of circumstances—while ironically implying an opposite (i.e., positive) appraisal. In the ironic criticism stimuli, speakers comment on a negative circumstance described in the short story (with a mock positive evaluation). In contrast, in the ironic praise stimuli, speakers comment on a positive circumstance (with a mock negative evaluation). In the TOVIDA-40, ironic utterances typically involve meta-messages indirectly implied by the speaker, such as when mocking the addressee's overly self-critical or self-effacing attitude^4^.

The scenarios are designed as ambiguous in order to warrant sufficient psychometric item difficulty, i.e., to avoid ceiling effects. This is why the stories still make some sense when irony is not detected in the ironic items (i.e., in the case of false negative detection) and when irony is falsely detected in the non-ironic items (i.e., in the case of false positive detection). Accounting for ambiguity in the process of irony detection, Utsumi (2000) points out that irony is distinguished from non-irony by assessing the degree to which a given utterance resembles prototypical irony. That is, not every ironic utterance unambiguously fulfills the constituting criteria of irony. Rather, the listener detects irony by assessing the similarity between a given utterance and a prototype of irony. Hence, ambiguity can be seen as a typical feature of real-life situations involving irony. However, in the scenarios of the TOVIDA-40 there are unobtrusive cues signaling the preconditions for the ironic utterance, i.e., hints to a reason for the speaker to express a negative attitude via ironic criticism or ironic praise (cf. Utsumi, 2000; Garmendia, 2014)^5^. In order to assess whether participants chose a literal or an ironic interpretation of target utterances, participants have to judge scenarios along statements about factual aspects of the situation or actors' emotional states as causes or consequences of target utterances. A person detecting the irony correctly appraises the situation differently from a person not detecting the irony. The TOVIDA-40 was designed as an unobtrusive test that can be optionally administered without any mention of irony and distracts test-takers from its true measurement intention. Six statements are provided for the appraisal of the situation (to be rated on a four-point scale ranging from 1 = “does not apply at all” to 4 = “fully applies”), among which three are indicative of irony detection (see Appendix). The other three appraisal statements are designed to distract from the intention of the task. For example, there is a statement asking whether the protagonists behave like a typical male or female (according to his or her gender) provided for every scenario. A high item score in the ironic items indicates correct positive irony detection, i.e., the comprehension of the true meaning of ironic target utterances as opposite to the literal meaning. The ironic items are administered alternating with the non-ironic distractor items.

#### Procedure

Participants were tested individually using an online-survey. They were randomly assigned to one of two groups (labeled here as Sample 1 and Sample 2). They either were instructed without any mention of irony (Sample 1: *irony non-alert* testing) or provided with a definition of verbal irony and instructed to watch out for irony in the stimuli, i.e., they were instructed that some of the scenarios they were about to appraise contain verbal irony whereas others do not (Sample 2: *irony alert* testing). Participants completed the TOVIDA-40 after they filled in questions about their demographic features and German language proficiency.

#### Preliminary analyses

In order to arrive at more reliable items scores, two of the three indicative statements were selected for every item applying a scale reliability criterion: inter-correlations between the three indicators were computed using Sample 1. The two indicators with the highest inter-correlation were selected and averaged to generate the item scores. In order to attain a more economic form of the TOVIDA-40, corrected item-total correlations (CITCs) were computed and considered as a selection criterion. Ironic criticism and ironic praise items were analyzed separately in this step. For selection purposes, only Sample 1 was used. For each of the two sub-scales eight items showed CITCs of r_cit_ ≥ 0.45 and were selected to build two scales to be analyzed in the further steps of Study 1. The 16 selected ironic items and the 10 non-ironic distractor items taken from the TOVIDA-40 will be referred to as the *TOVIDA* in the following sections.

### Results

Internal consistencies of the two resulting sub-scales were sufficiently high. Cronbach's alpha was 0.83 (0.76) for the ironic criticism scale and 0.83 (0.77) for ironic praise scale in Sample 1 and Sample 2 (values for Sample 2 in brackets).

**Figure 1:**
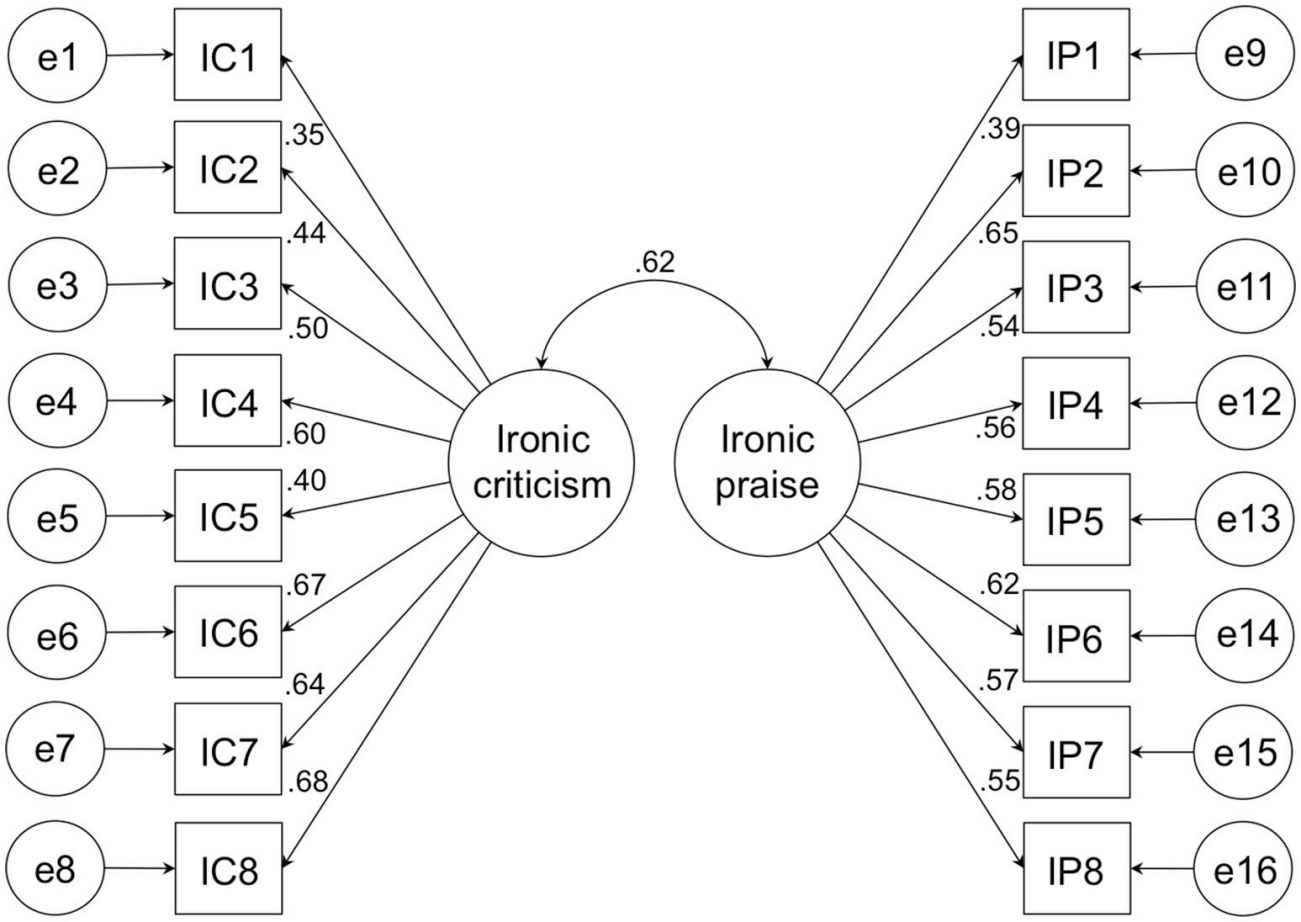
**Estimates for path coefficients in the two-factor model that was confirmed in Study 1**.

Within the irony alert sample (Sample 2), the fit of two different structural equation models was estimated. In the *assumed model*, two inter-correlating factors were modeled: one factor was defined by ironic criticism items and the other factor by ironic praise items. In the *control model*, a single factor was modeled defined by both ironic criticism and ironic praise items. As it turned out, the assumed two-component model had acceptable fit (c^2^ = 153.296, *df* = 103; Bentler Comparative Fit Index [CFI] = 0.906; root mean square error of approximation [RMSEA] = 0.056 [90% CI: 0.036; 0.073]; standardized root mean square residual [SRMR] = 0.0643). In contrast, the control model did not show acceptable model fit (χ^2^ = 227.025, *df* = 104; CFI = 0.771; RMSEA = 0.078 [90% CI: 0.071; 0.102]; SRMR = 0.0840). The path coefficients for the assumed two-factor model are given in Figure 1. As Figure 1 shows, the ironic criticism scale and the ironic praise factors were substantially intercorrelated.

### Discussion

The selection from the two types of items resulted in two scales with sufficient internal consistency. This indicates that there is an underlying irony detection aptitude creating shared variance in the items. Furthermore, the two-factorial structure could be affirmed, implying that ironic praise generated unique variance in the TOVIDA. Hence, the findings of Study 1 support the assumption that the aptitude to detect ironic praise is worth distinguishing from the aptitude to detect ironic criticism.

## Study 2: experimental evaluation of the TOVIDA

The stimuli employed in the TOVIDA were designed as ambiguous in order to warrant sufficient psychometric item difficulty, i.e., to avoid ceiling effects. Furthermore, irony detection is assessed indirectly in order to make a testing mode feasible in which subjects are non-alert to the occurrence of irony in the stimuli. So it was deemed necessary to validate that the stimuli of the TOVIDA truly contain irony, and if so, that high (vs. low) test scores truly indicate high (vs. low) irony detection performance. The aim of Study 2 was to address these questions.

Four criteria were defined to evaluate whether the TOVIDA allows for the assessment of irony detection: firstly, participants in the *irony alert* group are expected to have higher scores than participants in a *forced literal appraisal* group (i.e., participants instructed to view all items as non-ironic). This criterion reflects the consideration that there must be a group consensus among participants who know about the intention of the test that differs from a forced appraisal opposite to the designed ironic content. Secondly, participants in the *irony alert* group are expected to have higher scores than the ones in the *irony non-alert* group. The rationale of this criterion is that irony detection is facilitated when participants are instructed to watch out for irony (vs. being not informed about the possible occurrence of irony). Thirdly, a *forced ironic appraisal* group (i.e., participants instructed to view all items as ironic) is expected to have higher scores than the *forced literal appraisal* group. This criterion aims at ensuring that the appraisals used for the indirect measurement (and hence the item scores) are sensitive to irony detection. As a fourth criterion, the item scores within the *irony alert* group are expected to be positively correlated with direct appraisals (i.e., explicit ratings) of ironic content (these were assessed only in this group).

### Methods

#### Participants

Participants were recruited in university lectures, via university mailing lists, social platforms, and leaflets. The sample consisted of 154 German-speaking subjects (26 male [16.9%]). Participants' age ranged from 18 to 56 years with a mean of 24.8 years (*SD* = 7.8). They were randomly assigned to one of four testing conditions and the groups did not differ significantly as to age [*F*_(3, 150)_ = 1.69, *p* = 0.17], nor gender [*F*_(3, 150)_ = 0.085, *p* = 0.97].

#### Instruments

The *Test of Verbal Irony Detection Aptitude* (*TOVIDA; see* Study 1 for description and Appendix for an example item). Item scores were computed following the method of Study 1.

#### Procedure

In an online-survey, participants were randomly assigned to one of four test conditions: (1) one group was given a definition of verbal irony, was briefed that some of the scenarios they were about to see contain verbal irony whereas others do not, and instructed to take all target utterances as *ironic* when appraising the scenarios along the predefined statements (*forced ironic appraisal*), (2) one group was given a definition of verbal irony, was briefed that some of the scenarios they were about to see contain verbal irony whereas others do not, and instructed to take all target utterances as *literal* while appraising the scenarios (*forced literal appraisal*), (3) another group was given a definition of verbal irony, was briefed that some of the scenarios they were about to see contain verbal irony whereas others do not, and instructed to watch out for irony when appraising the scenarios according to their own interpretation (*irony alert*), and (4) the last group was instructed to appraise the scenarios according to their own interpretation without any mention of irony (*irony non-alert*). More specifically, the experimental instructions in the *forced ironic appraisal* group and the *forced literal appraisal* group briefed participants (a) to willfully view the last sentence in each of the situations as ironic or non-ironic, respectively, and (b) to respond to all of the concerned questions *as if* the last sentence was truly ironic or non-ironic, respectively. In the irony alert group, participants were requested to make direct appraisals (i.e., explicit ratings) of ironic content in addition to the standard appraisal. These explicit ratings of ironic content were assessed via a four-point Likert-type scale (1 = “not ironic,” 2 = “rather not ironic,” 3 = “rather ironic,” 4 = “ironic”), accounting for the ambiguous nature of the scenarios. Participants in the alert group were considered lay judges for this purpose (for the use of laypersons for validation purposes see Legree, 1995). The *irony alert* group was randomly over-sampled in order to warrant sufficient sample size for the planned correlational analyses.

### Results

#### Do the stimuli of the TOVIDA contain irony?

Group means of item scores are given in Table 1. As Table 1 shows, all items met the criterion to verify that they contain irony. More precisely, in line with the expectations, the *forced literal appraisal* group had lower means than the *irony alert* group with medium to large effect sizes, indicating that generally irony is detected in ironic items. Furthermore, in the *irony alert* group item scores were generally higher than in the *irony non-alert* group with small to large effect sizes (however, only 10 out of 16 of the comparisons yielded significant differences). In line with the expectation, being alert to irony facilitated irony detection.

**Table 1:** **Descriptive and test statistics of group scores in Study 2**.

**Item**	**Test instruction (group)**								**Group comparisons**					
	**1 Non-ironic**		**2 Ironic**		**3 Alert**		**4 Non-alert**		**1 vs. 2**		**1 vs. 3**		**3 vs. 4**	
	**M**	***SD***	**M**	***SD***	**M**	***SD***	**M**	***SD***	***t*_(63)_**	***d***	***t*_(90)_**	***d***	***t*_(87)_**	***d***
IC1	2.17	0.57	3.18	0.54	2.54	0.67	2.47	0.72	−7.25^*^	1.83	−2.59^*^	0.58	0.45	0.10
IC2	1.60	0.70	3.25	0.62	3.04	0.70	2.76	0.89	−10.09^*^	2.52	−9.06^*^	2.06	1.55	0.37
IC3	1.36	0.42	3.23	0.86	2.82	0.88	2.00	0.89	−10.63^*^	2.65	−8.34^*^	1.89	3.92^*^	0.93
IC4	1.37	0.55	3.50	0.72	2.64	1.05	2.13	1.06	−13.11^*^	3.26	−6.06^*^	1.37	2.05^*^	0.48
IC5	1.90	0.64	2.88	0.59	2.46	0.57	2.35	0.75	−6.35^*^	1.60	−4.19^*^	0.95	0.80	0.18
IC6	1.69	0.67	3.36	0.64	3.02	0.68	2.67	0.94	−10.23^*^	2.56	−8.62^*^	1.96	1.95	0.46
IC7	1.56	0.74	3.49	0.72	3.07	0.80	2.16	1.04	−10.52^*^	2.65	−8.54^*^	1.93	4.42^*^	1.04
IC8	2.08	0.81	3.25	0.67	2.89	0.56	2.43	0.82	−6.37^*^	1.60	−5.48^*^	1.26	3.03^*^	0.72
IP1	2.21	0.55	3.71	0.49	3.52	0.58	2.87	0.69	−11.59^*^	2.90	−10.13^*^	2.29	4.51^*^	1.06
IP2	1.57	0.84	3.31	0.77	3.18	0.61	2.67	0.98	−8.66^*^	2.17	−10.32^*^	2.34	2.96^*^	0.70
IP3	1.77	0.57	3.34	0.78	2.77	0.69	2.48	0.69	−8.93^*^	2.25	−6.66^*^	1.52	1.78	0.42
IP4	1.38	0.48	3.34	0.93	2.95	0.88	2.38	1.01	−10.18^*^	2.55	−8.88^*^	2.01	2.61^*^	0.62
IP5	2.01	0.52	3.05	0.69	2.94	0.51	2.57	0.54	−6.65^*^	1.67	−7.99^*^	1.81	3.02^*^	0.71
IP6	1.90	0.67	3.43	0.52	3.22	0.55	3.01	0.78	−10.39^*^	2.60	−9.86^*^	2.24	1.40	0.34
IP7	1.44	0.44	3.33	0.84	2.83	0.86	2.15	0.82	−10.87^*^	2.71	−8.09^*^	1.83	3.43^*^	0.80
IP8	1.77	0.67	3.00	0.91	2.60	0.70	2.21	0.57	−6.03^*^	1.51	−5.28^*^	1.20	2.47^*^	0.59

*IC1–IC8, ironic criticism items; IP1–IP8, ironic praise items. Non-ironic, forced literal appraisal (n = 28); Ironic, forced ironic appraisal (n = 37); Alert, irony alert testing (n = 64); Non-alert, irony non-alert testing (n = 25); d, Cohen's d coefficient of effect size*.

* *p < 0.05*.

Next, the direct appraisals of ironic content were examined to find out whether ironic items are viewed as more ironic than the non-ironic items. The frequencies of the single ratings were considered, given in Table 2. As Table 2 shows, ironic criticism items and the ironic praise items had numerically higher appraisals of being ironic (“rather ironic” and “ironic” answers) than non-ironic control items. It is noteworthy that the distributions of the proportions of ironic appraisals had a contact point: the ironic item with the lowest frequency of ironic appraisals (IC1) was judged about just as ironic as the non-ironic control item with the highest frequency of ironic appraisals (NC08). However, these two items can be seen as outliers in their group and as there was still a fair amount of judges consenting that the ironic items in question contain irony. Thus, they can be considered as difficult items but still containing irony. To test whether ironic criticism and ironic praise items were rated as more ironic than the non-ironic control items in the direct appraisals of ironic content, a mean of ratings over the eight items per scale was computed as well as the mean of ratings for the 10 non-ironic control items. These scores were compared with paired sample *t*-tests. It turned out that the non-ironic control items were rated as less ironic (*M* = 1.59, *SD* = 0.38) than the ironic criticism items [*M* = 2.95, *SD* = 0.58, *t*_(63)_ = −14.47, *p* < 0.001] and the ironic praise items [*M* = 3.23, *SD* = 0.54, *t*_(63)_ = −18.16, *p* < 0.001], indicating large effect sizes (i.e., *d* = 2.77 and *d* = 3.51, respectively)^6^.

**Table 2:** **Direct irony appraisal using explicit irony ratings for the single items of the TOVIDA (Study 2)**.

**Items**	**Rating scale steps**							
	**“Not ironic”**		**“Rather not ironic”**		**“Rather ironic”**		**“Ironic”**	
	***f***	**(%)**	***f***	**(%)**	***f***	**(%)**	***f***	**(%)**
IC1	19	(29.7)	19	(29.7)	12	(18.8)	14	(21.9)
IC2	3	(4.7)	6	(9.4)	22	(34.4)	33	(51.6)
IC3	6	(9.4)	17	(26.6)	15	(23.4)	26	(40.6)
IC4	15	(23.4)	10	(15.6)	17	(26.6)	22	(34.4)
IC5	10	(15.6)	16	(25.0)	20	(31.3)	18	(28.1)
IC6	7	(10.9)	3	(4.7)	25	(39.1)	29	(45.3)
IC7	6	(9.4)	8	(12.5)	17	(26.6)	33	(51.6)
IC8	5	(7.8)	13	(20.3)	14	(21.9)	32	(50.0)
IP1	5	(7.8)	6	(9.4)	8	(12.5)	45	(70.3)
IP2	3	(4.7)	2	(3.1)	4	(6.3)	55	(85.9)
IP3	10	(15.6)	20	(31.3)	16	(25.0)	18	(28.1)
IP4	7	(10.9)	5	(7.8)	23	(35.9)	29	(45.3)
IP5	2	(3.1)	3	(4.7)	25	(39.1)	34	(53.1)
IP6	2	(3.1)	3	(4.7)	13	(20.3)	46	(71.9)
IP7	8	(12.5)	12	(18.8)	14	(21.9)	30	(46.9)
IP8	11	(17.2)	12	(18.8)	23	(35.9)	18	(28.1)
NC01	33	(51.6)	21	(32.8)	7	(10.9)	3	(4.7)
NC02	49	(76.6)	14	(21.9)	1	(1.6)	0	(0)
NC03	34	(53.1)	18	(28.1)	4	(6.3)	8	(12.5)
NC04	37	(57.8)	20	(31.3)	2	(3.1)	5	(7.8)
NC05	47	(73.4)	9	(14.1)	6	(9.4)	2	(3.1)
NC06	34	(53.1)	23	(35.9)	6	(9.4)	1	(1.6)
NC07	56	(87.5)	6	(9.4)	1	(1.6)	1	(1.6)
NC08	18	(28.1)	20	(31.3)	13	(20.3)	13	(20.3)
NC09	46	(71.9)	15	(23.4)	2	(3.1)	1	(1.6)
NC10	34	(53.1)	19	(29.7)	8	(12.5)	3	(4.7)
**DESCRIPTIVE STATISTICS**								
*M*_IC_	8.88	13.9	11.50	18.0	17.75	27.7	25.88	40.4
*SD*_IC_	5.49	8.6	5.68	8.9	4.33	6.8	7.24	11.3
*M*_IP_	6.00	9.4	7.88	12.3	15.75	24.6	34.38	53.7
*SD*_IP_	3.55	5.5	6.27	9.8	7.55	11.8	13.41	20.9
*M*_NC_	38.80	60.6	16.50	25.8	5.00	7.8	3.70	5.8
*SD*_NC_	10.84	16.9	5.48	8.6	3.80	5.9	4.03	6.3

*N = 64. IC1–IC8, ironic criticism items; IP1-IP8, ironic praise items; NC01–NC10, non-ironic control items; M_IC_/ SD_IC_, mean/standard deviation for ironic criticism item ratings; M_IP_/SD_IP_, mean/standard deviation for ironic praise item ratings; M_NC_/SD_NC_, mean/standard deviation for non-ironic control item ratings*.

#### Is irony detection reflected in the item scores of the TOVIDA?

As Table 1 shows, the item score means of the *forced ironic appraisal* group were higher than item score means of the *forced literal appraisal* group, with large effect sizes. This indicates that a person will score high in all items if he or she detects the irony and score low if this is not the case. Finally, as expected, the direct appraisals (i.e., explicit ratings) of ironic content in the *irony alert* group correlated significantly with the respective item scores in all items with a mean of *r*_(63)_ = 0.72, indicating good convergence between direct and indirect appraisals. This finding indicates that the TOVIDA test scores reflect the degree to which participants considered the stimuli as ironic.

### Discussion

The results support the claim that, the ironic criticism and ironic praise stimuli used by the TOVIDA contain irony. Firstly, item scores were higher the group instructed to watch out for irony (i.e., the *irony alert* group) than in the group with experimentally induced minimal irony detection (i.e., in the *forced literal appraisal* group). This finding indicates that irony can generally be detected in the items of the TOVIDA (with a fair amount of interindividual variance, as shown by substantial standard deviations in *irony alert* and *irony-non alert* individuals' detection scores). Secondly, alertness to the ironic content of the stimuli fostered irony detection as the *irony-alert* group had higher item scores than the *irony non-alert* group in the majority of the items. Thirdly, the direct appraisals of the ironic content indicate that the ironic items were viewed as more ironic than the non-ironic items. There is also support for the claim that test scores reflect iron detection. Firstly, this was evident in terms of considerable differences between a group with experimentally induced minimal irony detection (i.e., in the *forced literal appraisal* group) and a group with experimentally induced maximal irony detection (i.e., in the *forced ironic appraisal* group). Secondly, the item scores corresponded well with direct appraisals (i.e., explicit ratings) of ironic content. These findings indicate that the items of the TOVIDA assess irony detection performance and that the stimuli—although they were designed as ambiguous—were consented as containing verbal irony to an acceptable degree.

## Study 3: exploring the usefulness of ironic praise in a study of irony detection correlates

Study 3 aimed at exploring whether ironic praise stimuli have a benefit in the investigation of ability and personality correlates of irony detection. Among the preexisting studies assuming an individual differences perspective in irony research, Ivanko et al. (2004) explored the possibility to explain interindividual variance in an irony interpretation task (i.e., in terms of participants' ratings of speaker's intent, such as *sarcasm, mocking*, and *politeness*) by means of participants' scores in “conversational indirectness” (i.e., the tendency to phrase one's remarks indirectly and the extent to which a person looks for indirect meanings in the remarks of others, cf. Holtgraves, 1997). The present study aims to extend this and other previous work (e.g., Blouin and McKelvie, 2012) by (a) looking at irony *detection* (rather than irony *comprehension* as the interpretation of speaker's attributes in ironic utterances) and (b) including intelligence and a broad range of personality traits as individual differences variables.

As one of the hypothesized correlates, it may be argued that trait cheerfulness has a relevance especially to the detection of ironic praise as cheerful individuals may have a more positive outlook on themselves and others and hence be more inclined to expect jolly and jovial interactions involving playful ironic teasing rather than hostile and negative interaction involving serious ridicule, such as in the form of ironic criticism. Furthermore, certain facets of the sense of humor may be more relevant to the detection of ironic praise than to the detection of ironic criticism. According to Ruch and Heintz (2016), the sense of humor includes also two virtue-related facets, i.e., *benevolent humor* and *corrective humor*. As an accepting way of dealing with negative circumstances (e.g., human weaknesses), benevolent humor may be relevant especially to ironic criticism (typically occurring in the face of negative circumstances) but not as relevant to ironic praise (typically occurring in the face of positive circumstances). That is, individuals prone to use, enjoy, seek, and understand benevolent humor may have a higher aptitude to detect ironic criticism. The other facet is characterized by tendencies to wittily ridicule those who deserve it from a moral stance in terms of *corrective humor*. Importantly, irony is listed as one of the ways in which corrective humor manifests itself in speech. It can be argued that by exposing transgressions of social rules in a witty and playful way, corrective humor is conceptually more related to ironic praise than to ironic criticism, which in turn can be seen as the more serious and less ingenious form of irony. Hence, individuals who are prone to use, enjoy, seek, and understand corrective humor, may have a higher readiness to detect irony in the case of ironic praise more than in the case of ironic criticism.

Furthermore, irony detection can be related to mental abilities—and presumably especially so in the case of ironic praise. According to previous studies (e.g., Mitchley et al., 1998) intelligence can be seen as a prerequisite for the detection of ironic criticism. Under the presupposition that the detection of ironic praise poses a different cognitive challenge to the individual than the detection of ironic criticism, there may be a unique relationship between the detection of ironic praise and mental abilities. Hence, a test for the assessment of general mental ability (i.e., intelligence) will be employed. To include a measure of an ability more specific to irony detection, a task by Winner et al. (1998) will be jointly administered that was designed to assess the ability to discriminate between irony and lies among patients with brain damage. Simultaneously, by testing its convergence with the detection of ironic criticism and ironic praise, the convergent validity of the TOVIDA will be explored.

Accordingly, we expect that there are associations between ironic praise detection and individual differences variables that are robust beyond the influence of the variance the detection of ironic praise shares with the detection of ironic criticism. Moreover, it is expected that both of the two scales of the TOVIDA correlate positively with the irony/lie discrimination task, as the ability to distinguish irony from a lie can be seen as relevant to ironic praise to the same extent as to ironic criticism.

As a secondary aim, the association between the two scales of the TOVIDA and the Big Five personality traits will be explored to learn more about the discriminant value of the irony detection measure. It is expected that the Big Five as broad personality dimensions distal to the sense of humor and distinct from mental ability are largely unrelated to irony detection scores. For exploratory purposes, again two testing modes will be employed with different degrees of *irony alertness*: hiding the measurement intention from participants (i.e., *irony non-alert* mode) vs. making irony salient (*irony alert* mode). As there are no comparable previous studies on personality and ability correlates of irony detection, it was preferred to include both the irony non-alert and the irony alert mode of testing in order to safeguard the investigation against a selective method bias.

### Methods

#### Participants

Participants were recruited in university lectures, and by means of university mailing lists, social platforms, and leaflets. Two independent quasi-experimental groups were tested. The first group (irony non-alert testing mode) consisted of 103 German-speaking subjects (28 male [22.0%]). Age in Group 1 ranged from 18 to 38 years with a mean of 21.6 (*SD* = 3.5). Group 2 (irony alert testing mode) consisted of 80 German-speaking subjects (16 males [17.6%]). Age in this group ranged from 18 to 46 years with a mean of 22.7 (*SD* = 5.5).

#### Instruments

##### Test of Verbal Irony Detection Aptitude (TOVIDA)

The *Test of Verbal Irony Detection Aptitude* (*TOVIDA*) was used for the assessment of irony detection performance (see Study 1 for description/Appendix). Item scores were computed following the method of Study 1. The scores of the eight ironic criticism items and the eight ironic praise items were averaged to build an ironic criticism detection score and an ironic praise detection score, respectively. The internal consistencies of the two scales were comparable to those found in Study 1. Cronbach's alpha was 0.81 (0.74) for the ironic criticism scale and 0.83 (0.79) for the ironic praise scale in the *irony non-alert* group and the *irony alert* group, respectively (values for the *irony alert* group in brackets).

##### Achievement Measurement System 2 (LPS-2 [Leistungsprüfsystem 2]; Kreuzpointner et al., 2013)

The LPS-2 is a performance test for the assessment of general mental ability. It employs 11 subtests that are allocated to four of the eight dimensions proposed by Carroll's (1993) model of intelligence, namely “crystallized intelligence” (e.g., solving anagrams), “fluid intelligence” (e.g., reasoning), “visual perception” (i.e., the ability to generate and process mental representations of spatial objects, to visualize, and to detect spatial patterns, e.g., mental rotation), and “cognitive speed” (e.g., arithmetic). A general IQ score is derived by aggregating the four subscales. Internal consistencies for subtests and the four dimensions are satisfactory in the norm sample with Cronbach's alpha ranging from 0.72 to 0.95. The total internal consistency for form A (form B) is high in the norm sample, α = 0.96 (α = 0.97). Split-half reliability of subtests ranges from sufficient (*r*_tt_ = 0.81) to high (*r*_tt_ = 0.93). Validity is confirmed in terms of concurrence with a range of other tests of mental ability. Furthermore, the targeted dimensional structure of the test is confirmed. The LPS-2 can be administered in groups and takes around 60 min to complete.

##### Irony/lie discrimination task (Winner et al., 1998)

This task measures the capacity to attribute second-order mental state and the ability to distinguish between ironic statements and lies. Subjects are required to read 15 short stories and to identify whether the final assertion is a lie or an ironic joke. There are eight stories involving a lie and seven stories implicating irony (in terms of intentionally and overtly uttering a counterfactual statement to a person known to be aware of the true circumstances). According to the characterization given by Winner et al. (1998), each story describes a context in which one person witnesses another individual breaking a rule sneakily (e.g., stealing food). The main difference between the two story types is that in the lie stories, the protagonist does not know that he or she had been seen doing the “sneaky action” and utters a lie to the witness to avoid getting caught. In the ironic stories, the protagonist knows he or she has been seen during the transgression and thereupon utters an ironic comment (i.e., a joke) to conceal his or her shame of being caught. For each story type (i.e., “joke” stories and lie stories), a separate score is generated by summing up participants' individual false negative decisions (i.e., the discrimination errors).

##### State-Trait Cheerfulness Inventory (STCI; Ruch et al., 1996)

The STCI is a questionnaire measure for the components of exhilaratability as the temperamental basis of the sense of humor. The trait version (STCI-T) encompasses three scales assessing *cheerfulness* (e.g., “I have a ‘sunny’ nature.”), *seriousness* (e.g., “I prefer people who communicate with deliberation and objectivity.”), and *bad mood* (e.g., “Even if there is no reason, I often feel ill-humored.”). In current study a 60-item short form of the STCI-T was used. The questionnaire assesses the endorsements of statements on a four-point scale (ranging from 1 = “strongly disagree” to 4 = “strongly agree”). Internal consistencies in the present sample were comparable to the ones in the construction sample reported by Ruch et al. (1996) with Cronbach's alpha ranging from 0.80 (seriousness) to 0.95 (bad mood).

##### Statements of Benevolent and Corrective Humor (BenCor; Ruch and Heintz, 2016)

The BenCor is a list of statements assessing two virtue-related facets of the sense of humor. *Six* statements are used for benevolent humor (e.g., “Even when facing unpleasant events I can keep my distance and discover something amusing or funny in it”) and corrective humor (e.g., “I caricature my fellow humans' wrongdoings in a funny way to gently urge them to change”), each. They were answered on a 7-point Likert scale ranging from 1 (“strongly disagree”) to 7 (“strongly agree”). Internal consistencies in the present sample were sufficient: Cronbach's alpha was 0.75 for benevolent humor and 0.78 for corrective humor.

##### Inventory of Minimal Redundant Scales

*Inventory of Minimal Redundant Scales* (*MRS-25* [Inventar Minimal Redundanter Skalen], Ostendorf, 1990; 25-item short form developed by Schallberger and Venetz, 1999). The MRS-25 is a list of 25 bipolar adjectives pairs for the assessment of the Big Five personality dimensions *extraversion* (e.g., impulsive vs. restrained), *agreeableness* (e.g., affirmative vs. oppositional), *conscientiousness* (e.g., diligent vs. lazy), *emotional stability* (e.g., robust vs. vulnerable), and *culture* (e.g., inventive vs. conventional). Answers are given on a six-point scale (*very—quite—rather—rather—quite—very*). Schallberger and Venetz (1999) report high internal consistencies of the scales and evidence for the validity of the MRS-25. Internal consistencies in the present sample were satisfactory with Cronbach's alpha ranging from 0.72 (agreeableness) to 0.86 (conscientiousness and emotional stability).

#### Procedure

Participants were tested in two consecutive sessions. In Session 1, groups up to 30 persons completed the LPS-2 as the first part of a larger assessment battery also including measures that were unrelated to the present study in the laboratory, quasi-randomly assigned to form A or Form B, depending on their seating position (as to avoid influence by neighboring participants). Due to time constraints, all other measures were included in an online survey. Participants were assigned an individual code and provided with an invitation containing an URL directing them to the online survey (Session 2). Within 7 days after Session 1, participants logged in and indicated their personal code for matching purposes. In Session 2, participants first completed the TOVIDA quasi-randomly assigned to one of two conditions: Half of the groups tested in Session 1 were given a definition of verbal irony and were instructed to watch out for irony, i.e., they were told that some of the scenarios they were about to appraise contain verbal irony whereas others do not (*irony alert* condition). The other half took the test naïve to its true intention (*irony non-alert* condition), i.e., there was no mention of the possible occurrence of verbal irony. Subsequently, STCI-T, the Big Five measure (MRS-25), the sense of humor measure (i.e., the BenCor), and the irony/lie discrimination task by Winner et al. (1998) were completed.

### Results

#### Is the detection of ironic criticism and ironic praise associated with abilities and traits?

The correlations between the two subscales of the TOVIDA and the other measures are given in Table 3, for the irony non-alert and the irony-alert group separately. As Table 3 shows, the ironic criticism scale was correlated substantially with the ironic praise scale but not correlated significantly with the other measures in the *irony non-alert* group. However, there was a trend for an association between the ironic criticism scale and the visual perception dimension of the LPS-2 (i.e., spatial ability), the performance in the ironic items (i.e., the joke stories) of the irony/lie discrimination task by Winner et al. (1998), and culture. In the *irony alert* group, again the ironic criticism scale was correlated substantially with the ironic praise scale. Furthermore, as expected, there was an association between the ironic criticism scale and the performance in the ironic items of the irony/lie discrimination task by Winner et al. (1998). Furthermore, there was also a trend for an association between the ironic criticism scale and emotional stability. In line with the expectations, among the self-report measures, bad mood and benevolent humor showed a significant relation to the ironic criticism scale and there was a trend for an association with cheerfulness. Furthermore, there was also a trend for ironic criticism detection showing an association with agreeableness and emotional stability.

**Table 3:** **Correlations between irony detection scores and the personality and ability measures (Study 3)**.

**Personality and ability measures**	**TOVIDA test instruction**					
	**Irony non-alert**			**Irony alert**		
	**IC**	**IP**	**IP_p_**	**IC**	**IP**	**IP_p_**
TOVIDA IP	0.52^*^	–	–	0.46^*^	–	–
**INTELLIGENCE (LPS-2)**						
Crystallized intelligence	0.03	0.04	0.03	0.00	0.10	0.11
Fluid intelligence	0.05	0.25^*^	0.26^*^	−0.04	0.17	0.21
Visual perception	0.15	0.16	0.09	0.07	0.28^*^	0.28^*^
Cognitive speed	−0.07	0.09	0.14	0.00	0.14	0.16
General IQ	0.06	0.22^*^	0.22^*^	0.03	0.23^*^	0.24^*^
**IRONY/LIE DISCRIMINATION**						
Irony (joke stories)	−0.17	−0.27^*^	−0.23^*^	−0.24^*^	−0.30^*^	−0.22
Non-irony (lie stories)	0.09	0.01	−0.04	0.10	0.02	−0.03
**BIG FIVE**						
Agreeableness	0.11	0.04	−0.02	0.17	0.02	−0.06
Conscientiousness	0.05	0.02	−0.01	0.05	0.12	0.12
Emotional stability	0.04	0.03	−0.01	0.17	0.24^*^	0.18
Extraversion	0.06	0.05	−0.03	0.01	0.17	0.20
Culture	0.14	0.19	0.14	0.04	0.01	0.03
**TEMPERAMENTAL TRAITS**						
Cheerfulness	−0.03	−0.08	−0.07	0.20	0.29^*^	0.23^*^
Seriousness	−0.08	−0.04	−0.01	−0.07	−0.04	−0.01
Bad mood	−0.03	0.01	0.04	−0.34^*^	−0.35^*^	−0.23^*^
**SENSE OF HUMOR**						
Benevolent humor	0.11	0.07	0.03	0.24^*^	0.30^*^	0.22
Corrective humor	−0.05	−0.06	−0.06	0.09	0.26^*^	0.25^*^

*n = 97–103 irony non-alert individuals. n = 80 irony alert individuals. IC, ironic criticism scale of the TOVIDA; IP, ironic praise scale of the TOVIDA; Sense of Humor, scales of the BenCor; IP_p_, partial correlations with IP controlling for the influence of IC*.

* *p < 0.05 (two-tailed)*.

As expected, the ironic praise scale was significantly correlated with intelligence in terms of fluid intelligence and with the performance in the ironic items of the irony/lie discrimination task in the *irony non-alert* group. Furthermore, there was a trend for an association with visual perception and culture for the ironic praise scale. In the *irony alert* group, the ironic praise scale was associated with intelligence in terms of the LPS-2 dimension visual perception (and there was also a trend for an association with the fluid intelligence dimension). Furthermore, the ironic praise scale again was negatively correlated with the number of errors made in the ironic items of the irony/lie discrimination task by Winner et al. (1998). Among the scales of the self-report measures, emotional stability, cheerfulness, bad mood, benevolent humor, and corrective humor showed significant correlations with the ironic praise scale. Furthermore, there was also a trend for an association with extraversion for the ironic praise scale in this group.

#### Are there unique correlates for ironic praise beyond ironic criticism?

Next, it was tested whether in the study of irony detection correlates ironic praise generates meaningful variance that contributes a surplus value over the meaningful variance found for ironic criticism. Therefore, partial correlations were computed between the ironic praise detection scale and the external variables while controlling for individuals' ironic criticism detection scores. The partial correlations are given in Table 3. As can be seen in Table 3, in the *irony non-alert* group, ironic praise correlated positively with *fluid intelligence* and negatively with the error rate in the irony items of the irony/lie discrimination task even beyond the influence of the variance shared with ironic criticism detection. In the *irony alert* group ironic praise correlated positively with the *visual perception* dimension of the intelligence test, trait cheerfulness, trait bad mood (in a negative direction), and corrective humor over and above the variance that the ironic criticism scale shared with ironic praise and these variables.

### Discussion

The findings of Study 3 indicate that assessing the detection of ironic praise can provide a surplus value over the detection of ironic criticism. Ironic praise detection can be seen as more challenging than the detection of ironic criticism in terms of numerically higher associations as well as significant partial correlations with the intelligence measure when the influence of the aptitude to detect ironic criticism was controlled for^7^. Hence, ironic praise detection appears to be dependent on mental ability to a certain degree, which is in line with previously reported findings on the role of intelligence in irony detection (e.g., Mitchley et al., 1998). However, considering the numerical size of the correlations, ironic praise detection aptitude can be seen as distinct from intelligence. Furthermore, as expected, it was found that the detection of ironic praise was uniquely associated with corrective humor, while ironic criticism was related only to benevolent humor. Also, cheerfulness played a unique role in the detection of ironic praise. Possibly increasing the readiness to process humorous meta-messages or playful cues in ironic teasing, a cheerful temperament hence can be assumed to facilitate the detection of irony, foremost in the form of ironic praise.

The Big Five personality traits were largely unrelated to irony detection scores except for a correlation between the ironic praise scale and *emotional stability*. It can be assumed that emotionally stable individuals have a higher readiness to reject the uttered criticism in what is literally said and recognize the more benevolent nature of what is ironically implied in the ironic praise items, compared to individuals low in emotional stability (who in turn may not “get over” the criticism or insult uttered in ironic praise). Although there was also a trend for an association between the irony detection scores on the one hand and culture and agreeableness on the other, the Big Five can be seen as less relevant for irony detection than narrower and more humor-related traits. Moreover, participants' scores in the TOVIDA converged with their scores in the ironic items of the irony/lie discrimination task, indicating convergent validity of the TOVIDA.

#### Do ability and personality variables interact in irony detection?

As an exploratory analysis complementing our correlational analyses, we wish to address the possibility that ability and personality variables interact in irony detection. To illustrate, although intelligence was found as positively related to irony detection, there might be highly intelligent individuals who still perform poorly in irony detection because they lack the requisite personality traits facilitating irony detection. Guided by the findings displayed in Table 3, we explored the data from Study 3 to see whether interactions between intelligence and personality could be found to predict irony detection beyond the main effects of the separate variables. Indeed, this assumption was found to hold true in one of the cases that we studied: in the irony-alert sample the interaction between the spatial ability dimension of the LPS-2 (i.e., *visual perception*) and *benevolent humor* predicted ironic praise detection significantly by explaining incremental variance beyond the main effects of the single predictors.

A hierarchical regression analysis with two steps was computed with the ironic praise detection score as the criterion. In Step 1, *visual perception* (β = 0.25) and *benevolent humor* (β = 0.26) were significant predictors, *F*_(2, 77)_ = 6.70, *p* = 0.002. As it turned out, the interaction term (computed as the simple multiplication of *visual perception* and *benevolent humor* scores) explained a significant increment of criterion variance when added to the equation in Step 2, *F*_(3, 76)_ = 7.27, *p* < 0.001; Δ*R*^2^ = 0.075, *p* = 0.008. As a possible interpretation of this finding, intelligence could be seen as a necessary but not sufficient condition for irony detection, as irony detection may be facilitated by individuals' cognitive ability only if individuals have enough sense of humor to successfully deal with irony. The inverse may also be true: the sense of humor may only manifest itself in irony detection performance if individuals have the necessary ability to successfully deal with its cognitive demands.

## General discussion

Our findings support the assumption that the detection of ironic criticism and the detection of ironic praise can be found as two intercorrelated but still discriminant facets of irony detection aptitude. Furthermore, our findings substantiate the assumption that ironic praise is useful beyond ironic criticism: applied in an investigation of ability and personality correlates, the detection of ironic praise was found to be uniquely associated with certain variables (i.e., intelligence, trait bad mood, trait cheerfulness, and the corrective facet of the measure of the sense of humor), beyond the influence of ironic criticism detection aptitude.

Extrapolating our findings, we may propose assumptions as to *why* more intelligent individuals high in cheerfulness and low in bad mood with high scores in benevolent and corrective humor may have a higher readiness to detect the irony in ironic praise. Maybe they are more able or ready to (a) reason and infer the meta-message of an ironic praise (i.e., *fluid intelligence*), (b) generate an easily interpreted mental “image” of the background of an ironic remark (i.e., *visual perception* as the ability to generate mental representations, to visualize, and to detect patterns), (c) take into account playful and humorous communicative intentions in terms of the processing of exhilarant stimuli (i.e., high trait cheerfulness and low trait bad mood), (d) have a smiling attitude toward the imperfections of life (e.g., human weakness) and know how to deal with them by using *benevolent humor* (i.e., in terms of the principle “it takes one to know one”), and (e) expose transgressions of morally valued social rules by using irony with satirical meta-messages in order to educate and better social others (i.e., the tendency to produce, to enjoy, and to make sense of *corrective humor*).

### The role of irony alertness

There was an irregularity in the findings of Study 3 (which, however, occurred in a quite constant fashion): in the *irony non-alert* group, the association between the personality variables and irony detection was not evident compared to the *irony alert* group. As a possible explanation for this finding, participants in the *irony non-alert* sample may have been biased toward expecting a bona fide communication mode, as in the given psychological assessment situation a serious state of mind may have been induced. This consideration may have an implication for the assessment of irony detection in general terms, as in many of the pre-existing measurement procedures for the assessment of irony detection irony alertness is reduced by not mentioning to participants that the stimuli they are about to encounter contain irony and by using indirect measurement (i.e., not asking the participants directly whether they think that there is irony in a stimulus)^8^. At least as far as the study of personality and ability correlates of irony detection is concerned, it can be seen as worthwhile to further explore the benefit of maximizing irony alertness and using direct testing.

### Is the TOVIDA too difficult?

In the construction of the TOVIDA we assumed that, in order to tap into the variance in irony performance among normally functioning adults, psychometrically difficult items need to be employed (as to avoid ceiling effects). Notably, there is a trade-off between item difficulty (i.e., ambiguousness of the stimuli) and test-takers' consensus as to the ironic nature of the stimuli. Certainly, the items should not be too difficult to allow for a sufficient consensus among test takers as to whether irony is present in the stimuli or not. However, a fair amount of variance (i.e., an imperfect consensus) can be argued to be admissible as this variance (a) must be expected when conceptualizing irony detection aptitude as an approximately normally distributed variable, and (b) is rooted in the nature of the construct when dealing with phenomena involving an inherent uncertainty, which—apart from irony—can also be found for example in certain knowledge domains. Accordingly, Legree (1995) for example argues in favor of a Likert-based assessment of social intelligence because of the level of uncertainty involved in the stimuli. He characterizes the challenge of assessing knowledge of ambiguous relationships when he states that “situational judgment scales attempt to simulate everyday problem situations but cannot allow the formulation of unambiguously “correct” solutions. This ambiguity partially reflects real-world interpersonal interactions, which are often ambiguous […]” (Legree, 1995, p. 249).

### The possible role of self-involvement

In the TOVIDA, test-takers have to make sense of situations containing verbal irony from an observer's perspective (i.e., with low *self-involvement*). It would also be thinkable to test irony detection performance using self-involving situations, such as when instructing test takers to place themselves into the respective situation as if they would encounter them in real life. Importantly, this may lead to certain variables coming into play more prominently as correlates of irony detection performance. For example, self-involvement may accentuate the association between ironic praise detection and *emotional stability*. If a specific instance of ironic praise is an interpersonal evaluation, emotionally unstable individuals may be more attached to the negative interpersonal valence of the verbatim utterance (which can occur in the form of a mock critical offense) and hence may be less prone to reject the literal interpretation of the ironic remark—and importantly so this mechanism may be accentuated as self-involvement in the assessment of irony detection increases. This consideration may also apply to certain other traits, such as self-esteem or the fear of being laughed at (i.e., gelotophobia; cf. Ruch et al., 2014). For example, because of their general belief to be inherently ridiculous and deficient, gelotophobes may be sensitive to derisive ironic criticism especially when self-involvement is high. Accordingly, future studies investigating traits relevant to derisive criticism or offense in irony detection should explore the benefit of self-involving test stimuli and instructions.

## Conclusions

Ironic criticism and ironic praise can be seen as separate scales in irony detection. The two types of irony were differently related to ability and personality variables, as ironic praise detection showed unique associations with intelligence and certain traits. Hence,—at least as far as the stimuli used in our investigation are concerned—ironic praise can be postulated to generate variance with surplus meaning beyond the variance generated by ironic criticism in irony detection. Consequently, ironic praise as the less “prototypical” and formerly neglected type of irony and can be postulated as especially important to include when studying the role of ability, personality, and humor in irony detection.

## Ethics statement

This study was carried out in accordance with the recommendations of the Psychological Research Ethics Committee of the University of Zurich with written informed consent from all subjects. All subjects gave written informed consent in accordance with the Declaration of Helsinki. The protocol was approved by the Psychological Research Ethics Committee of the University of Zurich.

## Author contributions

RB: Data collection, data analysis, drafting manuscript. WR: Data analysis, drafting manuscript.

### Conflict of interest statement

The authors declare that the research was conducted in the absence of any commercial or financial relationships that could be construed as a potential conflict of interest. The reviewer UB and handling Editor declared their shared affiliation, and the handling Editor states that the process nevertheless met the standards of a fair and objective review.

## Footnotes

^1^To illustrate: imagine that a circle of friends is watching a sports match and some of the attendees support Team A while other attendees support Team B. An example of ironic criticism would be if one of the supporters of Team A said “Terrific shot! You're handing us a resounding defeat!” when a player of Team B tries but fails to score a goal in the match (for example when the speaker wants to ridicule the arrogant prediction made by one of the supporters of Team B that “their” Team B would win at a canter). In contrast, if one of the supporters of Team A said “Terrible shot! We don't stand the slightest chance!” when a player of Team A scores a goal, this would be an example of ironic praise (for example when the speaker wants to ridicule one of the supporters of Team B for his or her arrogant prediction that Team A would lose the match in a sad spectacle of defeat).

^2^The terms “irony” and “sarcasm” are sometimes used interchangeably (e.g., Pexman and Olineck, 2002) and there is an ongoing debate as to whether sarcasm and irony are essentially the same thing (cf. Attardo, 2000). However, we wish to adhere to a demarcation between irony and sarcasm in terms of two naturally overlapping but conceptually distinct phenomena. For example, in the *Merriam-Webster Dictionary* sarcasm is defined as “a sharp and often satirical or ironic utterance designed to cut or give pain.” Irony can be seen as related to sarcasm because phrasing a criticism ironically was found to enhance the degree of perceived condemnation (as compared to phrasing it literally, i.e., Colston, 1997). In the present paper we will stick to the term *irony* (even if sarcasm is involved in a specific instance of irony), foremost because if any of the studies in our literature review use the term *sarcasm*, they originally refer to *ironic* sarcasm (rather than *non-ironic* sarcasm).

^3^Langdon et al. (2002) use the term *banter* when labeling the category of their stimuli containing a negative statement being used in a positive context (which corresponds to the definition of ironic praise we adhere to) without the intention to harm or to criticize, whereas their *sarcasm* stimuli were characterized by a positive statement used in a negative context (which corresponds to the definition of ironic criticism) with the intention to harm or to criticize. It is necessary to mention here that elsewhere bantering irony was conceptualized to occur not only in situations in which the speaker intends to ironically praise (i.e., in terms of *kind banter*) but also when *ironically criticizing* (i.e., in terms of *sarcastic banter*; cf. Anolli et al., 2002). However, as far as we can tell from their report, Langdon et al. (2002) used banter only in the form of *kind banter* (i.e., they did not include *sarcastic banter* involving ironic criticism) in their banter stimuli.

^4^As speakers say something different from what they actually want to express, irony classifies as an indirect speech act, cf. (Holtgraves, 1997). What makes irony different from other forms of indirect speech acts is that ironic speech acts are characterized by an overt insincerity. That is, ironic speakers achieve indirectness by engaging in an evident dissimulation when inversing the valence of their true appraisal in the verbatim utterance (i.e., especially by using a choice of words denoting *the opposite* of their true appraisal of circumstances, cf. Attardo, 2000, 2001).

^5^As Garmendia (2014) argues, irony is always negative in terms of a critical attitude. That is, also in the case of ironic praise, which—as a meta-message—can be described to typically involve a hint to the transgression of (sometimes unwritten) rules, for example the norm of not to be vain, not to boast, not to be arrogant, not to be overly modest, not to make false promises, and so on. That does not mean that there is not another meta-message on a higher level that can be characterized as benevolent and more positive. For example, ironic teasing can be corrective and bonding at the same time, as the teaser implies that he or she thinks that the relationship with the teased person is strong and close enough to make playful provocation possible without risking a serious social damage (cf. Norrick, 1994; Boxer and Cortés-Conde, 1997).

^6^An exploratory analysis indicated that ironic praise items were appraised as somewhat more ironic than the ironic criticism items with a medium effect size (*d* = 0.50). This is important to point out, as the direct appraisals are substantially correlated with the item scores (i.e., the indirect appraisals). The irony in ironic praise items hence can be seen as less difficult to detect than ironic criticism. It is not clear whether this is owed to the fact that the ironic praise items used in the present set of studies are less ambiguous than the ironic criticism items or whether ironic praise *per-se* is easier to detect than ironic criticism.

^7^Differential associations between the two scales of the TOVIDA and the intelligence variables could be explained by differences in average item difficulty. As ironic praise items were more frequently appraised as ironic than ironic criticism items in Study 2, it is possible that the lack of association between the ironic criticism scale and intelligence hence might be an artifact created by higher ambiguity of the materials. This may be the case because intelligence may foster irony detection only when items have a low ambiguity.

^8^In previous studies indirect measurement of irony detection was operationalized for example by resorting to fact questions (e.g., Ackerman, 1983; Happé, 1993), using questions targeting mental states of the speaker and emotions of the target of the ironic utterance (e.g., McDonald and Pearce, 1996), asking whether it made any sense for the speaker to make the target utterance (e.g., Langdon and Coltheart, 2004), or rewording the use of irony as “joking” (e.g., Winner et al., 1998).

## Appendix

The following sample item of the TOVIDA given below is translated from German language. The statements for the appraisal of the situation were rated on a four-point scale as to how much the sentences apply to the situation (1 = “does not apply at all,” 2 = “rather does not apply,” 3 = “rather applies,” 4 = “fully applies”). Statements printed in bold were used as (inversed) indicators of irony detection in the studies and averaged to build the item score. Three of the appraisal statements were designed as distractors for each item.

### Ironic praise sample item (IP6)

Situation:

Christian has invited three friends over for dinner. He prepares a meal trying a new recipe. Sitting at the table starting to eat, Julia asks for the saltshaker. Christian immediately apologizes that he could not salt the food to taste as he has a cold and cannot taste properly. This is when Julia says: “You are right, the food is inedible!”

Instruction: *Please indicate how much you think that each of the following statements applies to the situation*.

Statements:

1. *The saltshaker is not within Julia's reach*.
2. *Christian behaves like a typical male*.
3. *Julia is having a good time*.
4. ***Christian will feel bad because of Julia's final utterance. (***−***)***
5. ***Julia will not finish her plate without more salt. (***−***)***
6. *Christian is making up excuses*.
